# First Chromosome-Scale Assembly and Deep Floral-Bud Transcriptome of a Male Kiwifruit

**DOI:** 10.3389/fgene.2022.852161

**Published:** 2022-05-16

**Authors:** Jibran Tahir, Ross Crowhurst, Simon Deroles, Elena Hilario, Cecilia Deng, Robert Schaffer, Liam Le Lievre, Cyril Brendolise, David Chagné, Susan E. Gardiner, Mareike Knaebel, Andrew Catanach, John McCallum, Paul Datson, Susan Thomson, Lynette R. Brownfield, Simona Nardozza, Sarah M. Pilkington

**Affiliations:** ^1^ The New Zealand Institute for Plant and Food Research Limited, Auckland, New Zealand; ^2^ Department of Biochemistry, University of Otago, Dunedin, New Zealand; ^3^ The New Zealand Institute for Plant and Food Research Limited, Manawatu Mail Centre, Palmerston North, New Zealand; ^4^ The New Zealand Institute for Plant and Food Research Limited, Lincoln, New Zealand

**Keywords:** male kiwifruit genome, sex determining region, long read genome assembly, transcriptomic of male flower, PacBio and illumina sequencing, male—genetics, whole-genome sequencing (WGS)

## Introduction

Over 50 kiwifruit species from the genus *Actinidia* represent climbing perennial vines that show variable hardiness and dioecy and produce fruits with a delicious flavor (Kawagoe and Suzuki, 2004). Yet, very few genotypes are commercialized (Huang et al., 2000; Ferguson and Huang, 2007). With its center of origin and distribution in south-central and southeast China (Huang, 2016), kiwifruit was originally referred to as “Monkey Peach” or Mihoutao. High vitamin C content (McCallum et al., 2019) combined with exquisitely diverse flavors and pigment contents (Zhang et al., 2017) are among the top breeding targets (Webby et al., 1994; Laing et al., 2007; Ampomah-Dwamena et al., 2009; Montefiori et al., 2011) for today’s billion-dollar kiwifruit industry.

Natural variation in chromosomal copy numbers in *Actinidia* is remarkable with a range of different ploidy levels (*2x*, *4x*, *6x*, *8x*, and *10x*) across species (Ferguson and Huang, 2007). Cultivars from three *Actinidia* taxa are well established in the global fruit markets: *A. chinensis* var. *deliciosa* (green kiwifruit), followed by *A. chinensis* var*. chinensis* (yellow- and red-fleshed kiwifruit), and *A. arguta* (often called kiwiberry) (Ferguson and Huang, 2007; Latocha, 2017). The genetic diversity of the genus *Actinidia* is, however, poorly explored. Being dioecious, that is, separate male and female plants, genomes of kiwifruit cultivars show high heterozygosity (Huang et al., 2013), which significantly influence the breeding of commercial kiwifruit as higher ploidy alone is not responsible for elite traits in kiwifruit cultivars (Li et al., 2010; Zhang et al., 2017). In the last decade, two accessions from *A. chinensis* var. *chinensis* (‘Hongyang’/HY, Red5 v1) and one from *A. eriantha* (‘White’) have been assembled (Huang et al., 2013; Pilkington et al., 2018; Tang et al., 2019; Wu et al., 2019) using advanced combinatorial approaches in stitching complex genomes together. A draft genome assembly of *A. rufa* ‘Fuchu’ has also been developed with 647.2 Mb and scaffold-N50 of 15.9 Mb (Kagawa, 2020). Where these genome assemblies have been progressively improved with higher scaffold-N50 lengths (23.5 Mb) (Tang et al., 2019), they represent a collapsed view of the heterozygous diploid state of the genome (*2n*) so they lack resolution at the haplotig level (*n*). The four currently available whole-genome assemblies of *Actinidia* species further represent only females with little information on the landscape of the sex-determining region (SDR) on the Y chromosome of *A. chinensis*. It is known that the sex-determining genes in *Actinidia* are tightly linked to the upper arm of the linkage group 25 (Akagi et al., 2018; Akagi et al., 2019); however, the genomic view of this landscape is fragmented.

In this study, we present the first-ever view of the chromosomal-scale assembly of the male diploid *A. chinensis* var. *chinensis* accession named ‘Russell’, previously referred as P1 (Tahir et al., 2019) which shows high tolerance to bacterial canker *Pseudomonas syringae* pv. *actinidiae* biovar 3 (Psa). We have also performed deep transcriptome analysis of the immature tissues of floral buds to capture the differentially expressed genes in developing anthers and aborting carpel in male flowers and expression of the sex-determining genes. This dataset will help advance our understanding of kiwifruit genetics, evolution of sex, plant development, and disease resistance.

## Data Briefs

Our approach for whole-genome assembly (Supplementary Figure S1) involved Single Molecule Real-Time (SMRT) sequencing technology to generate long-read data with paired and mate-paired-end Illumina sequencing to develop primary and haplotig genome assemblies and genetic mapping to validate the ordering of scaffolds. This process yielded a final primary assembly containing 618.6 Mb assigned to 29 linkage groups with a scaffold-N50 of 21.77 Mb. Contigs identified as haplotigs included 424 contigs containing 372.6 Mb with N50 of ∼1.3 Mb and minimum/maximum lengths of 1.53 kb and 6.15 Mb, respectively. The basic metrics of the final assembly units (primary, haplotig, and unassigned) are provided in Supplementary Table S1. Locations of the haplotigs relative to the final primary assembly are presented in Supplementary Table S2. Of the linkage group assignments, eight (chr1, chr5, chr8, chr10, chr17, chr19, chr24, and chr25) showed terminal telomeric repeat regions at both their 5’ and 3’ termini, while for 20 linkage group assemblies (chr2, chr3, chr4, chr6, chr7, chr9, chr11, chr12, chr13, chr14, chr15, chr16, chr18, chr20, chr21, chr22, chr23, chr27, chr28, chr29), evidence of a terminal telomeric repeat region was found at only one of these or other termini. One linkage group (chr26) did not have evidence for telomeric repeat regions at either terminus.

To estimate genome completeness, Benchmarking Universal Single-Copy Orthologs (BUSCO) (Simão et al., 2015) analysis was performed on the primary assembly and the haplotigs dataset separately. There were 95.9% complete BUSCOs in the primary assembly sequence dataset with 72.3% being single copies and 23.6% being duplicated. The analysis indicated that 1.1% of BUSCOs were fragmented while 3.0% were missing. BUSCO evaluation of the haplotigs dataset identified 1,123 (69.6%) complete BUSCOs of which 58.6% were single copies and 11.0% duplicated. There was a higher level of fragmented BUSCOs (2.9%) in haplotigs and 27.5% of BUSCOs were missing. The latter result was expected since contigs in the haplotig set represent alternate assembly units for only parts of the primary assembly genome sequence.

Previous *Actinidia* genome assemblies have highlighted the density of repeat regions in the genome (up to 43%), courtesy of long terminal repeats (LTRs), as well as heterozygosity based on the occurrence of 17-mers. We performed *de novo* transposable element (TE) detection on the ‘Russell’ genome (Supplementary Table S3). The abundance of the annotated TEs across chromosomes is presented in Supplementary Figure S2. Around 41.47% of the ‘Russell’ genome was annotated as TEs. The percentage of bases masked on each chromosome ranged from 30.52% on chr23 to 53.13% on chr27, while chromosomes with the least and most abundant TE loci were chr4 (15,875 TEs) and chr8 (29,894 TEs), respectively (Supplementary Table S4). Mutator and helitron were the two most abundant TE types, with an average of 4,812 and 3,483 sites per chromosome, followed by LTR/Copia (2,919), repeat region (2,737), general LTR (2,667), hAT (2,498), and LTR/Gypsy (2,399).

For annotation, we employed homology-based alignment of existing gene models coupled with manual curation review using RNA-Seq evidence. This yielded 33,833 gene models in the primary assembly, and 20,533 were located on haplotig assembly units, yielding 54,366 gene models. The metrics are presented in Supplementary Table S5. The number of new or revised models created per assembly unit (linkage group) is presented in Supplementary Table S6. Of these models, 42 were identified as partial sequences, with 17 being truncated at their 3 prime termini (no stop codon) and 25 lacking a start codon, among primary and haplotig assembly models.

**FIGURE 1:**
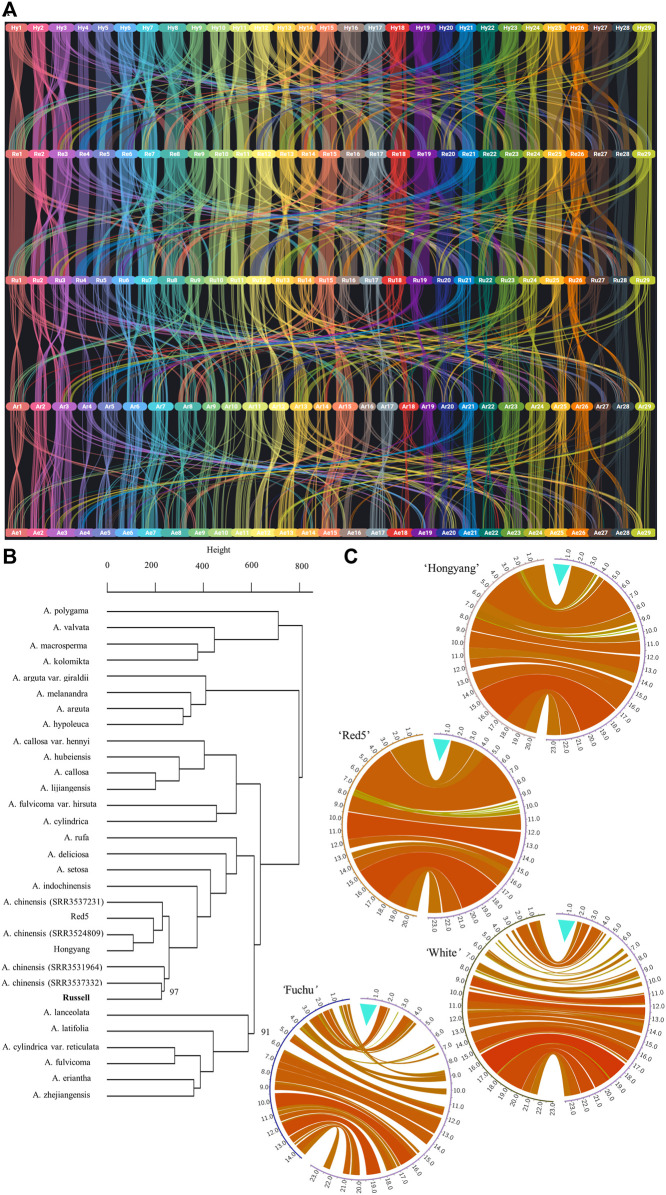
Comparison of the ‘Russell’ genome with other *Actinidia* genomes. **(A)** shows synteny for protein-encoding genes, across the genomes, as they are anchored on respective chromosomes 1-29 in the form of blocks. The synteny as well as inter-chromosomal homology or duplication events, described by co-linearity metrics drawn from BLASTP and MCScanX, for protein-encoding genes. The genomes are in the following order from top to bottom: *A. chinensis* ‘Hongyang’ (v3), *A. chinensis* Red5 (v2), *A. chinensis* ‘Russell’, *A. rufa* ‘Fuchu’ and *A. eriantha* ‘White’. Two letter codes were applied as follows: Hy—‘Hongyang’, Re—Red5, Ru—‘Russell’, Ar—*A. rufa* and Ae—*A. eriantha*. ‘Hongyang’ chromosome assemblies were renamed to conform to the linkage group numbers used across the other whole-genome assemblies. **(B)** A bootstrapped dendrogram based on 1,000,000 sampled variants called from coding sequence within publicly available whole-genome sequence data mapped to the ‘Russell’ genome assembly. All nodes gave bootstrap values of 100%, except for nodes marked otherwise as percentages. Clustering shows broad support for clade groupings. The height between nodes represents the distance between clusters of the respective nodes. **(C)** shows pairwise DNA alignment (using nucmer) between chromosome 25 of the male *A. chinensis* var. *chinensis* ‘Russell’ and chromosome 25 from four female *Actinidia* whole-genome sequences. The pairwise comparisons were with ‘Hongyang’ version 3, Red5 version 2, *A. rufa* “Fuchu’ and *A. eriantha* ‘White’. In all comparisons, chromosome 25 from ‘Russell’ is depicted on the right hand side. The turquoise colored mark indicates the male-only first 1.5 Mb region on the sex chromosome. The relatedness between the genomic sequence of each genome pair is depicted by a combination of the width of any individual circos plot “ribbon” (the wider the ribbon the longer the region of genome similarity) and the color used for the ribbon where ribbon color (yellow to red) reflects increasing numbers of links aggregated into bundles using the parameters described in the methods with the unitary scale in circos equivalent to 1 Mb. In all comparisons, the first 1.5 Mb of the ‘Russell’ chromosome has no alignment to any other region of chromosome 25 (nor to any other chromosome) in any of the five female genomes. This region contains the sex determining genes, only expressed in the early stage of anther and carpel tissues.

Protein-block synteny based on predicted proteins from the male ‘Russell’ assembly and all-female whole-genome assemblies for *Actinidia* species was examined. Figure 1A shows a higher view of protein block synteny among *A. chinensis* genomes *Actinidia chinensis* ‘Hongyang’ (v3) (Wu et al., 2019), *A. chinensis* Red5 (v2) (Pilkington et al., 2021), *A. chinensis* ‘Russell’, compared to either *A. rufa* ‘Fuchu’ (Kagawa, 2020) or *A. eriantha* ‘White’ (Tang et al., 2019). To investigate the evolutionary relationship between ‘Russell’ and the publicly available whole-genome sequencing data for kiwifruit in Sequence Read Archive (SRA), we generated a bootstrapped dendrogram of 31 samples of 18 *Actinidia* species (Figure 1B). Information on the Sequence Reads Archive dataset for *Actinidia* species is present in Supplementary Table S7. The dendrogram closely resembles the species distribution of gene trees developed previously (Liu et al., 2017). As expected, it indicates that ‘Russell’, being *A. chinensis* var. *chinensis*, shows little partition from ‘Hongyang’ and Red5, with *A. rufa* closest to *A. chinensis* clade.

To validate the assembly of the Y-chromosome we aligned 249 *de novo* assembled DNA Y-specific contigs, described previously (Akagi et al., 2018), to the ‘Russell’ whole genome. Out of 249, 216 of the contigs aligned with chr25 (Supplementary Table S8). On ‘Russell’ chr25, the male-specific contigs identified previously (Akagi et al., 2018) align essentially in two clusters. The first cluster containing 179 contigs begins at position 2,363 bp and extends through to position 1,462,475 bp. This region shares little homology to the published female *Actinidia* whole genome sequences (Pilkington et al., 2018; Tang et al., 2019; Wu et al., 2019; Pilkington et al., 2021) and is presented in Figure 1C and Supplementary Figure S3. BLAT (Kent, 2002) alignment of the complete coding sequences for the two Y-chromosomes, specifically sex encoding genes, *Shy Girl* (LC260493.1) (Akagi et al., 2018) and *Friendly Boy* (LC482704.1) (Akagi et al., 2019), showed both to be completely present on chr25 of ‘Russell’ and expressed in the floral tissues analyzed for RNA-Seq. The *Shy Girl* locus (RUSV2a.044824.1.PC) was found to be located in the reverse orientation on chr25 between positions 6,906 and 9,504 while *Friendly Boy* (RUSV2a.044829.1.PC) was located between positions 701,402 and 703,048. This places *Shy girl* and *Friendly Boy* approximately 687 Kb apart on the proximal end of chr25 of ‘Russell’. This 687 Kb region is a part of the longer ∼1.46 Mb cluster described previously. The second cluster on ‘Russell’ chr25 includes 18 Y-specific contig alignments and is aligned to a 79 Kb region that extends from positions 4,950,289 bp to 5,029,529 bp.

**FIGURE 2:**
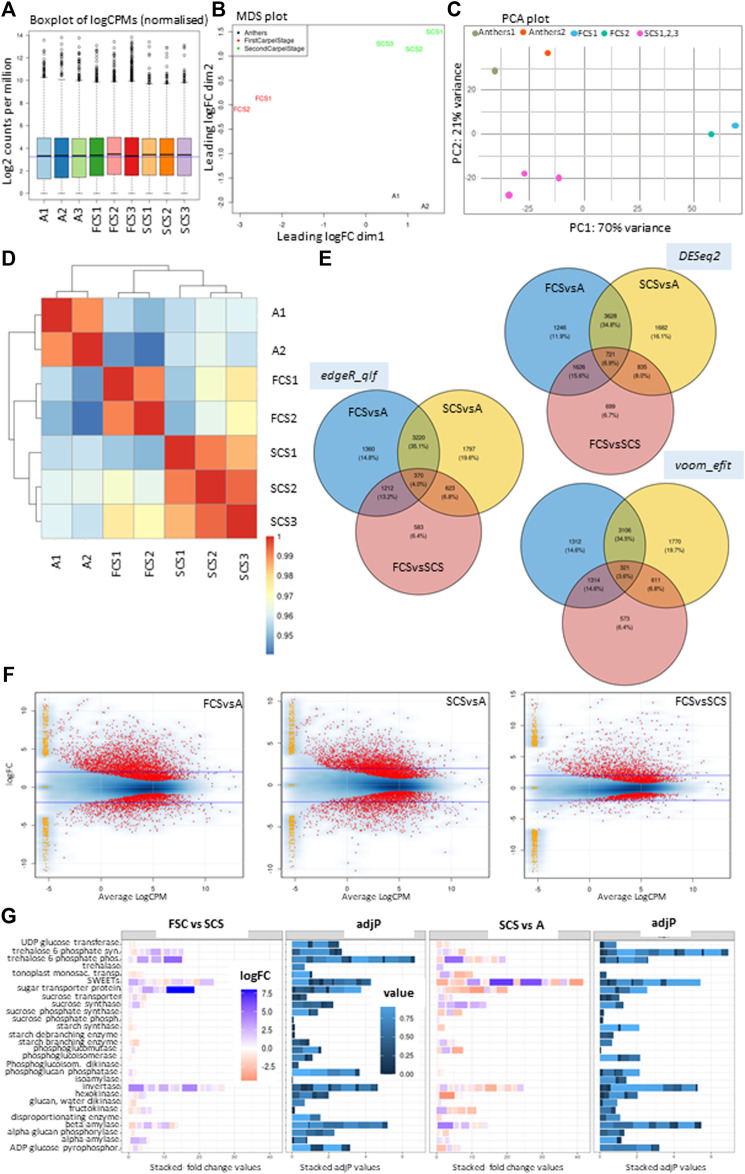
Differential expression (DE) analysis of the transcriptomes of *Actinidia chinensis* var. *chinensis* Russell’s immature anther and carpel tissues. **(A)** shows the boxplot of logCPMs of normalised libraries for all samples, **(B)** shows multidimensional scaling analysis plot (MDS) for the samples selected for DE analysis, **(C)** shows the Principal component analysis, and **(D)** shows the heatmap clustering of all the log-transformed raw counts from samples selected. **(E)** shows Venn diagram for DE genes between three tissues, using three independent methods including edgeR (test-qlf, FDR < 0.05), DESeq2 (padj <0.05) and voom (test-efit, padj <0.05). **(F)** shows smearplots for DE genes, highlighted in red at *p* value ≤0.01 with a blue line marked at logFoldChange (logFC) ±2, in a pair-wise analysis of tissues of First Carpel Stage (FCS) vs. Anther (FCSvsA), Second Carpel Stage (SCS) vs. Anther (SCSvsA) and First Carpel Stage vs. Second Carpel Stage (FCSvsSCS) using edgeR. The yellow-orange dots placed on the left side of the plots are showing logFC in genes that are uniquely expressed in each tissue with a total count of zero for the other group as provided in the smear plotting function, i.e., adding the “smear” of yellow points at low value towards the left side of the graph. The width is adjusted by random uniform numbers of width “smearWidth”. **(G)** shows fold changes (stacked for each enzyme class as bars) and corresponding adjP values (stacked as bars for enzyme class with fold changes described) for major gene families involved in carbon metabolism within carpel tissue (FCS versus SCS) and between tissues (SCS versus Anthers).The scale for fold changes and adjP values are provided in respective boxes.

To dissect the transcriptomic changes in male floral parts, we performed deep RNA-seq of the tissues of the immature floral components that were dissected at stages 1 and 2, as described previously (Akagi et al., 2018; Akagi et al., 2019) i.e., immature anther tissues (A), harvested from Stage 2, whereas carpels were harvested from Stage 1 and 2 and referred to as First Carpel Stage (FCS) and Second Carpel Stage (SCS) (Figure 2A). The raw counts and sample information are provided in Supplementary Table S9. A multi-dimension analysis and PCA plot show that biological replicates were nicely clustered together (Figures 2B and C). One of the samples from FSC and Anthers was removed from the analysis considering higher to moderate dispersion in PCA plots. Differential expression (DE) analysis with and without these excluded samples is provided in Supplementary Data S1. The heatmap clustering further shows that samples from two carpel stages are closer to each other compared to anthers (Figure 2D). DE analysis was performed using three independent methods including voom (Law et al., 2014), edgeR (Robinson et al., 2010), and DESeq2 (Love et al., 2014) (padj < 0.05). DE genes between tissues and a method-wise analysis are presented in Figure 2E and the corresponding dataset is provided in Supplementary Data S1. It shows that around 4–6.9% of the total differentially expressed genes (DEGs) in all the pair-wise analyses among tissues are shared across methods. Secondly, among all the three pair-wise comparisons, 11.9–14.8% of DEGs are unique to AvsFCS, 16.7–19.6% are unique in AvsSCS while 6.4–6.7% are unique to FCSvsSCS. It is noticeable that 34.5–35.1% of the DEGs are in common when Anthers were compared with either of the carpel stages, FCS or SCS. Contrastingly, fewer DEGs are found to be shared when one of the Anther vs. Carpel stages is compared with the Carpel vs. Carpel stage i.e., 13.2–15.6% in between AvsFCS: FCSvsSCS and 6.8–8% in between AvsSCS: FCSvsSCS. In terms of the number of DEGs obtained in each pair-wise analysis using edgeR (FDR <0.05 and fold-change ±1.2); 1) 4176 genes were up-regulated while 1986 were down-regulated in FCS as compared to Anthers, 2) 3866 genes were up-regulated and 2148 were down-regulated in SCS compared to Anthers and 3) 1969 genes were up-regulated and 819 genes were down-regulated in FCS compared to SCS. The smearplots (Robinson et al., 2010) (Figure 2F) further show the density of genes uniquely expressed in each tissue.

We compared differential expression for the major gene families involved in carbon metabolism (Nardozza et al., 2020) within carpel tissue (FCS versus SCS) and between tissues (SCS versus Anthers) Figure 2G (corresponding dataset in Supplementary Table S10). A comparison between FCS and SCS samples highlights that the key genes involved in sucrose cleavage are invertases (group *alpha cytosolic invertases*, *INVA* and *INVE,* and *apoplastic cell wall invertase CWINV2* and *sucrose synthase SUSA2.p*). These sucrose-cleaving genes are up-regulated early in carpel development, suggesting a decline in sucrose cleavage as an increase in *Shy Girl* expression starts to suppress carpel development (Liao et al., 2020). It is worth noting this is the first time that *CWINV2* expression has been detected in kiwifruit, as it is not expressed in later stages of fruit development (Nardozza et al., 2013) and represents a key gene for apoplastic unloading. At stage 2, anthers represent a strong sink compared with carpels, as suggested by high sucrose synthase transcription. With the exception of up-regulation of *BAM3.2/BAM3.3* and *BAM4.1*, starch synthesis and degradation are not very different between the two tissues, suggesting they are not storing reserves: carpels are being suppressed, whilst anthers are in a growing phase.

The resistance response against the bacterial canker caused by Psa biovar 3 from ‘Russell’ (P1) (Tahir et al., 2019) is found to be polygenic and hence the durable form of resistance relies on gene families likely involved in basal defense. Cell walls become the first line of defense and play a crucial role in a plant’s basal resistance (Malinovsky et al., 2014). *Cellulose synthase-like* (*CSL*) genes govern cellulose synthesis and a gene from this family (Acc15562.1) was found to be differentially induced in the Psa-tolerant F1 progeny of ‘Russell’ (Tahir et al., 2019). We performed a comparative assessment of the family of *Cellulose synthase-like* (*CSL*) genes in the ‘Russell’ genome, with the manually annotated Red5 (Pilkington et al., 2018), which includes *CESA, CSLA, CSLB, CSLC, CSLD CSLE* and *CSLG* class of genes. A total of 58 models were identified from both Red5 and ‘Russell’, from which eight new models were predicted from the ‘Russell’ genome, suggesting a 14% improvement compared with the previous Red5 genome (Supplementary Tables S11, 12). The consensus tree from the alignment of these genes shows how sub-groups of various classes of genes form part of the CES-like family (Supplementary Figure S4; Supplementary Table S12). The complete genome view of ‘Russell’ will provide more insight into gene families which provide durable resistance.

## Materials and Methods

### Plant Source, DNA, and RNA Extraction

*Actinidia chinensis* (Planch.) var. *chinensis* ‘Russell’ was originally collected in 1981 as seed from the wild, in Liuyan County of the Hunan province of China (M. McNeilage, pers. comm.) and has been grown in various research and commercial orchards. The nuclear genomic DNA for ‘Russell’ was extracted, from leaves collected from orchard-grown vines, with cetyltrimethylammonium bromide (CTAB)-based buffer as described previously (Naim et al., 2012). Total RNA from leaves was extracted using the CTAB-LiCl method (White et al., 2008). Immature floral buds from ‘Russell’ canes were dissected at Stage 1 and 2, as described previously (Akagi et al., 2018; Akagi et al., 2019). Immature anther tissues (A), which are black, were harvested from Stage 2, whereas carpels were harvested from Stage 1 and 2 and referred to as First Carpel Stage (FCS) and Second Carpel Stage (SCS) samples. RNA extraction was performed using Spectrum™ Plant Total RNA Kit, Sigma-Aldrich (Darmstadt, Germany). Total RNA had high yields (25–40 µg per 100 µL per extraction) and quality (RQN 7.3–9.6 by Fragment Analyser; Agilent, United States). A total of nine samples, were sent for sequencing to the Australian Genome Research Facility (Melbourne, Victoria Australia).

### Genome and RNA Sequencing

Pacific BioSciences (Menlo Park, California, United States) Sequel Single Molecule Real-Time (SMRT) sequencing technology was used to generate long-read data from ‘Russell’ nuclear DNA for whole-genome assembly at the Genetics Laboratory, Central Analytical Research Facility, Queensland University of Technology, Brisbane, Australia. Fasta sequences for subreads were extracted from subread bam files from 13 SMRT cells using bam2fasta from bam2fastx, which is part of the PacBio® tools distributed *via* Bioconda (Grüning et al., 2018). A total of 6,262,602 subreads were extracted, which collectively possessed an N50 of 31,803 bases: the longest subread length was 244,154 bases, and the shortest subread was 50 bases and the mean and median subread lengths were 19,726 and 16,438 bases, respectively. The raw subreads from these 13 cells had approximately 163x coverage assuming a genome size of 758 Mb. In addition, high-quality Illumina short-read sequencing data were also generated containing ∼181 Gb of paired-end reads (125 bp size) and ∼150 Gb of mate-paired end libraries with three different insert-sizes, 3–5, 8–10, and 11–15 kb. Nine Illumina total RNA stranded libraries, three from each of Russell’s A, FSC, and SCS tissues were prepared and sequenced using NovaSeq® S4 Lane generating a 618.86 Gb total dataset, with each sample tissue represented by ∼100 million, 100 bp paired-end reads. RNA from ‘Russell’ leaf samples was sequenced using NovaSeq, generating ∼12 Gb of 150 bp paired-end reads.

### Genome Assembly

The subread fasta from SMRT was assembled using FALCON and FALCON-UNZIP (Chin et al., 2013; Chin et al., 2016). The assembly contigs were filtered using the “trimLowercaseContigs.py” (https://github.com/PacificBiosciences/apps-scripts) to cull contigs. To remove any Smartbell adapters, the primary and haplotig contigs were re-merged into a single multiple fasta file and adapter removal was performed using “removesmartbell.sh” from BBMap (Bushnell, 2014). The use of removesmartbell.sh and “split = t” yielded 2,243 contigs. Reads from Nextera mated pair libraries with average insert size ranges of 3–5, 8–10, and 11–15 kb were trimmed using NxTrim (O’Connell et al., 2015) (version 0.4.2). Unique read pairs for each library were extracted using an in-house PERL script yielding 121,177,199, 177,136,753, and 56,569,541 read pairs, respectively, for each of the three libraries. The mated pair libraries were then used to scaffold the long-read assembly contigs using SSPACE_basic_v2.0 (Boetzer et al., 2010). Contigs were then analysed with Purge Haplotigs (Roach et al., 2018) (version 1.1.0-0) using setting “LOW = 32 MID = 136 HIGH = 232” to re-sort contigs into primary and haploid contig sets. Prior to running the minimap2 alignment (Li, 2018) step in Purge Haplotigs, all assembly input subreads were corrected using the corrector from Canu (Koren et al., 2017) (version 1.9) and 735,117,238 Illumina 125 base paired-end reads pre-trimmed by 10 nucleotides at each terminus. The outputs from purge haplotigs were subject to gap closer using Abyss-sealer (Paulino et al., 2015) (version 2.1.5) to seal gaps of 3,000 bases or less (“--max-gap-length = 3000”) using the same trimmed paired-end reads data as used for correction of long reads. Primary contigs were assigned to linkage groups using information from a genetic map previously described (Pilkington et al., 2018) with additional input from DNA-based synteny to other whole genome sequences for *Actinidia* species as follows: DNA markers (Pilkington et al., 2018) were aligned with the ‘Russell’ contigs using Bowtie2 (Langmead and Salzberg, 2012) (version 2.3.4.3). Chromonomer (Catchen et al., 2020) (version 1.09) was then used to develop initial placement. Placement of contigs for which no markers exist was based on the consensus of DNA synteny-based comparison to published *Actinidia* genomes (Pilkington et al., 2018; Tang et al., 2019; Wu et al., 2019) and to the long-read-based genome assembly for Red5 V2 (Pilkington et al., 2021). Synteny was also employed to inform re-ordering of contig placements.

Methods for 1) gene annotation using a hybrid approach and de novo TE detection, 2) naming convention for gene IDs, 3) comparative genomics and phylogenetic analysis, and 4) transcriptomic of immature floral components are provided in Datasheet 1 Supplementary Methods.

## Data Availability

Russell genome (Primary assembly, Haplotig assembly, unassigned scaffolds and Supplementary Data S1 (RNA-Seq data analysis) is available online at Figshare https://figshare.com/s/c6ffcc5300533022cc5a. The raw dataset, including reads for genomic and rna datasets, is available at SRA BioProject: PRJNA811574: First chromosome-scale assembly and deep floral-bud transcriptome of a male kiwifruit, Sequence Read Archive (SRA) submission: SUB11128479 https://www.ncbi.nlm.nih.gov/sra/PRJNA811574.
