# Supplementary material for: First Chromosome-Scale Assembly and Deep Floral-Bud Transcriptome of a Male Kiwifruit
Source: Front Genet. 2022 May 16;13:852161. doi: 10.3389/fgene.2022.852161 (PMC9149279; doi:10.3389/fgene.2022.852161)
Supplement: Supplementary file 1 [file DataSheet1.zip › Supplementary Figures.DOCX]

Supplementary Material

**Supplementary Figures**

Supplementary Figure 1. Generalised schematic of whole genome assembly approach for ‘Russell’.

Supplementary Figure 2. Transposon element abundance across chromosomes. Plot shows count and the density of distinct TEs genome-wide in *Actinidia chinensis* var. *chinensis* ‘Russell’.

Supplementary Figure 3. Circos plot of the pairwise DNA alignment between chromosome 25 of the male *A. chinensis* var. *chinensis* ‘Russell’ and chromosome 25 from Red5 version1.

Supplementary Figure 4. Tree from the alignment of all the CES-like genes containing the GT domain.


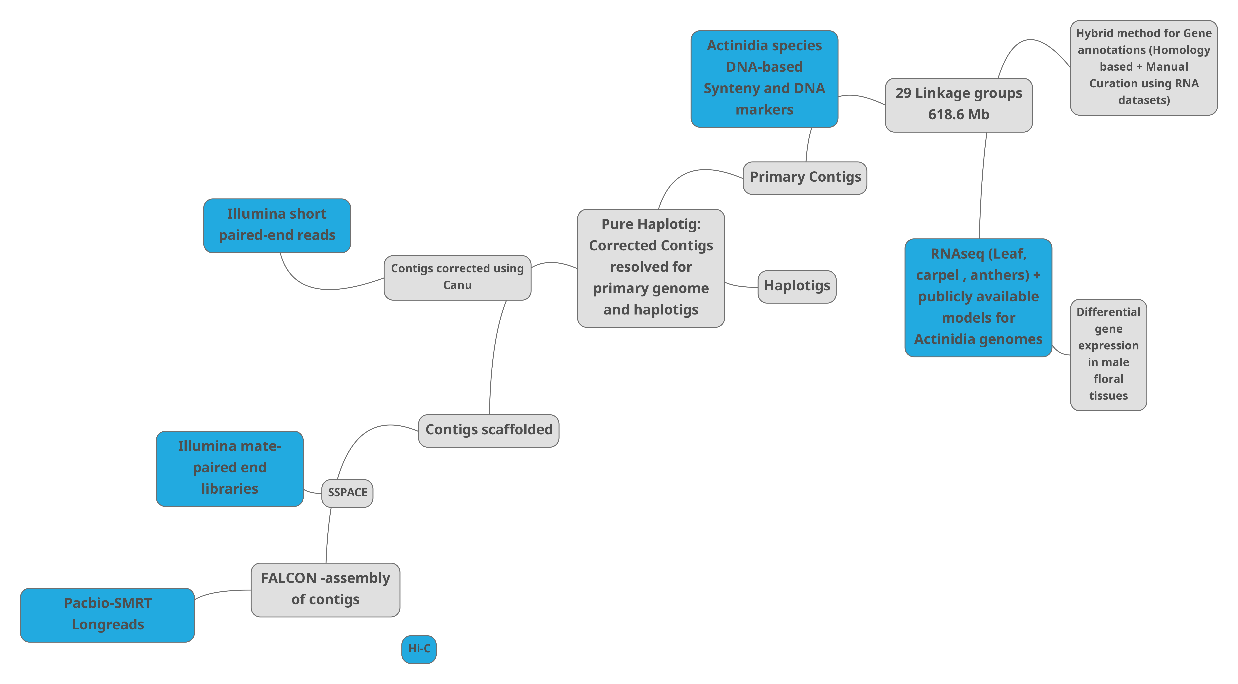


**Supplementary Figure 1.** Generalised schematic of whole genome assembly approach for ‘Russell’.


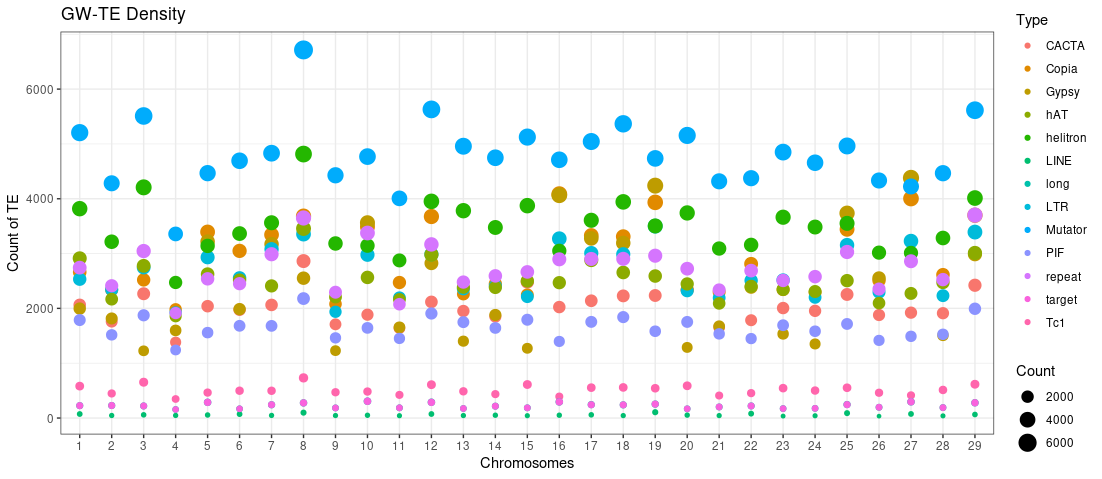


**Supplementary Figure 2.** Transposon element abundance across chromosomes. Plot shows count and the density of distinct TEs genome-wide in *Actinidia chinensis* var. *chinensis* ‘Russell’.


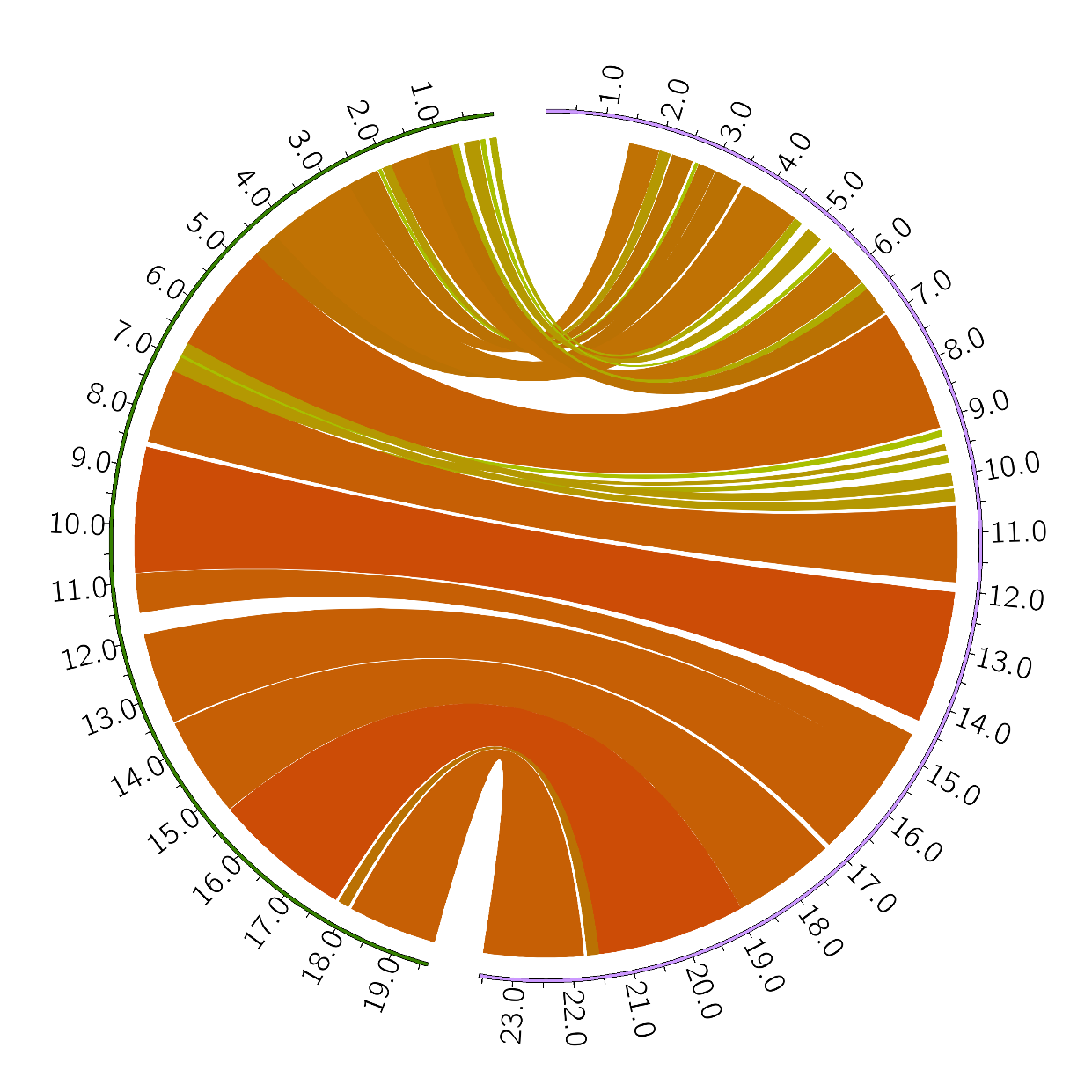


**Supplementary Figure 3**. Circos plot of the pairwise DNA alignment between chromosome 25 of the male *A. chinensis* var. *chinensis* ‘Russell’ and chromosome 25 from Red5 version1.


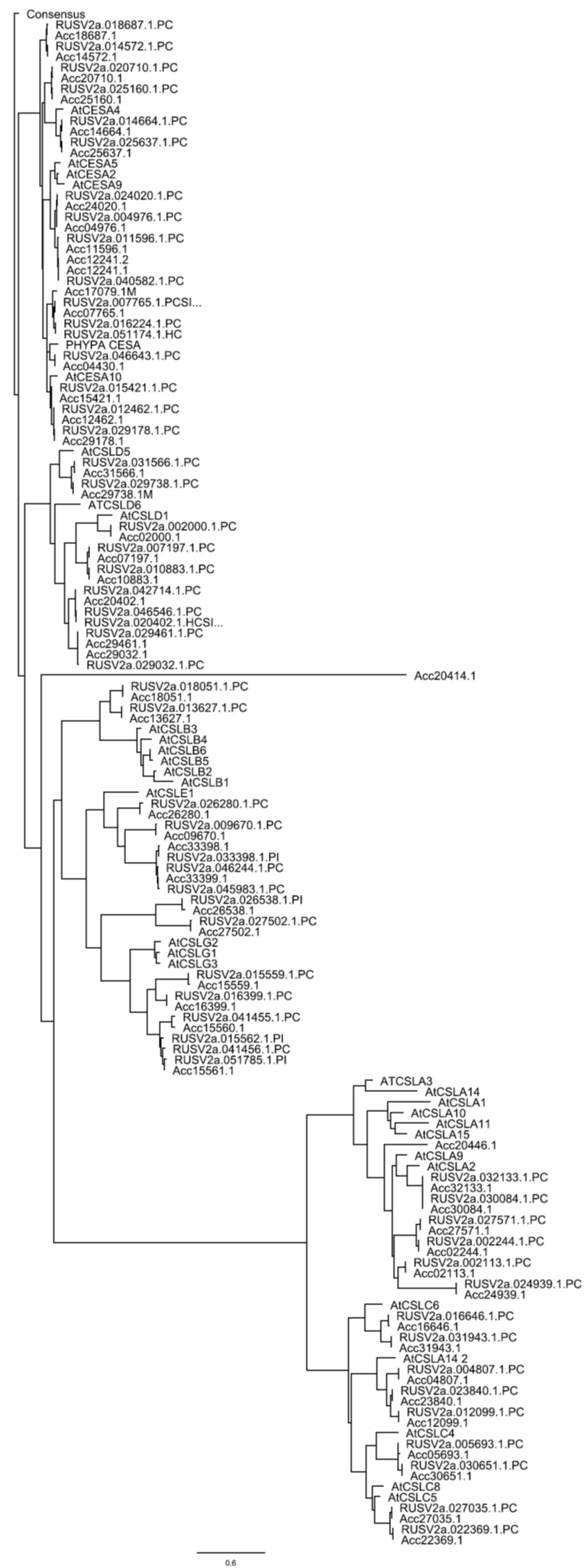


**Supplementary Figure 4**. Tree from the alignment of all the CES-like genes containing the GT domain.
