# Supplementary material for: First Chromosome-Scale Assembly and Deep Floral-Bud Transcriptome of a Male Kiwifruit
Source: Front Genet. 2022 May 16;13:852161. doi: 10.3389/fgene.2022.852161 (PMC9149279; doi:10.3389/fgene.2022.852161)
Supplement: Supplementary file 1 [file DataSheet1.zip › Supplementary Methods.DOCX]

Supplementary Methods

# Gene annotation using hybrid approach and de novo TE detection

The primary assembly (linkage group) fasta was uploaded to a local instance of the gene annotation tool WebApollo2 (Dunn et al., 2019) (version 2.0.6). The gene models from *Actinidia chinensis* var. *chinensis* Red5 (v1) (Pilkington et al., 2018), *A. eriantha* ‘White’ (Tang et al., 2019) and *A. rufa* ‘Fuchu’ (NCBI Bioproject PRJDB8483)(PRJDB8483, 2020) were downloaded from NCBI (accession numbers NKQK01000000, QOVS00000000, and BJWL01000001, respectively). The gene models for *A. chinensis* var. *chinensis* ‘Hongyang’ (v3) (Wu et al., 2019) and Red5 (v2) (Pilkington et al., 2018) were obtained from Sequence Read Archive database (Bioproject PRJNA54977, GenBank assembly accession GCA_009663005.1) and Zenodo (accession 10.5281/zenodo.5717387) respectively. Gene models from the four genomes above were aligned to the whole genome sequence of ‘Russell’ using GMAP (Wu and Watanabe, 2005) and alignments with at least 90% coverage and 90% identity retained. The resulting gff3 alignment files were uploaded to WebApollo2 as separate tracks to use as annotation guides. RNA-Seq reads generated from ‘Russell’ leaves and publically available RNA-Seq reads from Red5 (Pilkington et al., 2018) and ‘Hongyang’ (SRR3923798, SRR3923799, SRR392800, SRR392801, SRR392803, and SRR392804) were aligned to the whole genome sequence of ‘Russell’ using STAR (Dobin et al., 2012) (version 2.6.1d) and made available inside WebApollo2 as sorted bam files. ESTs from *A. chinensis* var. *chinensis* and PacBIO RS II full length RNA sequences from adult female leaves (SRX7090265) were downloaded from NCBI Genbank and aligned with GMAP to the ‘Russell’ whole genome sequence as well as the de novo assembled transcript set from ‘Russell’ RNA-Seq dataset. Prior to use, de novo assembled transcript sets were clustered using cd-hit-est (https://github.com/weizhongli/cdhit/wiki) using ‘-c 0.98’. The resulting alignments were made available as evidence tracks in WebApollo2 where manual curation of the primary assembly was performed. The manually curated models were then exported from WebApollo2 and the exported gff3 merged with alignments for Red5 models (those directly accepted as described above). The program gffread (https://github.com/gpertea/gffread) was then used with command line options (‘-i 94000 -C -A --sort--alpha -F -P --adj-stop --merge -K -Q -D -S’) to extract CDS (CoDing Sequence), cDNA and predicted protein sequence from the ‘Russell’ whole genome sequence using the merged gff3 as a guide. In order to identify gene models on haplotigs, the cDNA and CDS sequences for the ‘Russell’ models from linkage group sequences were aligned to the haplotig assembly contigs using GMAP. These models were then compared with NCBI RefSeq Release 203 (plant subset) using BLASTp and the results evaluated to identify models deemed to be internal fragments relative to the target database. This led to the removal of 1,215 models. All models with predicted protein sequence lengths of less than 25 amino acids were removed from the annotation sets. This threshold was somewhat arbitrarily based on the minimum length of photosystem II protein L proteins (26 amino acids) found in RefSeq 203 (plant subset). The program ‘agat_sp_filter_incomplete_gene_coding_models.pl’ from AGAT (https://github.com/NBISweden/AGAT) was used to assess partialness of models. All the metrics (Supplementary Table 1) were extracted from assemblies using a modified version of the assemblathon_stats.pl program (https://github.com/KorfLab/Assemblathon/blob/master/assemblathon_stats.pl ) using command line options ‘-n 1 -genome_size 758000000’.

We performed de novo transposable element (TE) detection on the ‘Russell’ with the Extensive De novo Transposable Element Annotator (EDTA) (Su et al., 2021) (v1.9.3). The pipeline incorporated a list of repeat detection packages (such as LTR_Finder, LTRharvest, HelitronScanner, TIR-Learner, RepeatModeler, and LTR_retriever) and yielded a consensus result. The input parameters specified to EDTA were“--species others --step all --sensitive 1 --anno 1 --evaluate 1 --threads 20”).

# Naming convention for gene IDs

Models names were assigned using the following naming convention: <genome assembly prefix>.<numeric identifier>.<transcript number>.<molecule class suffix> where the ‘genome assembly prefix’ was RUSV2a and the ‘molecule class suffix’ was defined as follows: ‘PC’ for primary assembly model deemed complete (having both start and stop); ‘PI’ for primary assembly model deemed to be incomplete (truncated at one or other terminus); ‘HC’ for haplotig assembly model deemed complete (having both start and stop); ‘HI’ for haplotig assembly models deemed to be incomplete (truncated at one or other terminus). The numeric identifier for each model was based on the following: where best reciprocal BLASTp analysis to the manually curated gene set for Red5 identified a 1:1 best reciprocal blast relationship (taking into account chromosomal location for non-single copy models) the numeric component following the prefix ‘Acc’ of the original Red5 was applied to the ‘Russell’ model. The transcript number was set to 1. When there was more than one ‘Russell’ model with equal best BLASTp then each was assigned the numeric integer but the transcript number was incremented sequentially. Where no relationship existed by best reciprocal blast, or was ambiguous with more than two ‘Russell’ models sharing the same BLASTp bitscore and expect, then a new numeric value was assigned.

# Comparative genomics and phylogenetic analysis

Synteny of our ‘Russell’ whole genome sequence to those in published whole genome sequences from *Actinidia* species was examined in two ways. In the first approach, DNA:DNA sequence alignment was used to examine homology between ‘Hongyang’, Red5, and ‘Russell’. Each genome was compared pairwise using nucmer (Marçais et al., 2018) and the alignments summarised with dnadiff (Marçais et al., 2018). The resulting 1coords file was converted to Circos (Krzywinski et al., 2009) links and then links bundled using bundlelinks from the Circos Tools package (http://circos.ca/software/download/) using command line options ‘-max_gap 20000 -min_bundle_membership 5’. Following bundling, ribbons were colorized based on the number of links in the bundle. Additionally, we explored protein-block synteny based on predicted proteins from the male ‘Russell’ assembly and all female whole genome assemblies for *Actinidia* species including those for *A. rufa* and *A. eriantha*. Pairwise best reciprocal BLASTp analysis results were filtered to remove alignments with less than 50% coverage and 80% identity. Pairwise results were then merged into a single BLASTp results file. Positional locations were extracted from the gene model gff3 available for each genome and merged into a single file in the format required by MCScanX (Wang et al., 2012). MCScanX analysis was performed using these files and command line options (‘-s 5 -m 25’). The resulting multi-genome gff and collinearity files were then visualised using SynVisio (Bandi, 2020) to provide an upper level view of protein-blocks synteny genome-wide.

From the publicly available whole genome sequencing (WGS) data for kiwifruit in Sequence Read Archive (SRA), we generated a dendrogram of 31 samples of 18 *Actinidia* species (Figure 1B) by alignment to the ‘Russell’ genome assembly using BWA-MEM (Li and Durbin, 2009) (v. 0.7.17). Variants were called using freebayes-parallel (Garrison and Marth, 2012) (v. 1.1.0). Genotypes for construction of the dendrogram were filtered to include only those of coding sequence (using the gff file for ‘Russell’), and were extracted into a “012” file using vcftools (Danecek et al., 2011), where 0 indicates homozygous reference, 1 indicates heterozygous, 2 represents homozygous alternate, and -1 represents a null allele. Variants with any samples that contained null alleles were discarded from the analysis. A sample of 1000,000 variant sites was used as data to generate a bootstrapped (1000 times) dendrogram with the R package pvclust, using the “complete” method of hierarchical clustering and the “euclidean” method of distance measure.

*Cellulose synthase-like* (*CSL*) gene family phylogeny tree (Supplementary Figure 4) was generated using the methodology previously described in Pilkington et al. (2018).

# Transcriptomic of immature floral components

We performed RNA-Seq of immature floral components, Anthers, FCS and SCS tissues, with three biological replicates each tissue. Fastq files for all samples were assessed for quality using FastQC and adaptors were trimmed from all samples using Trimmomatic (Bolger et al., 2014) with the following parameters (illuminaclip:(clip):2:30:10 headcrop:12 slidingwindow:5:20 minlen:36 leading:3 trailing:3). Reads were aligned to the ‘Russell’ genome using HISAT2 (Kim et al., 2019) and SAM/BAM files (sorted) were generated using SAMtools (Li et al., 2009) (version 1.12). The alignment rate to the whole genome (primary, haplotigs and unassigned) and primary genome ranged between 84 and 81%, respectively. Ribosomal content of the reads was also assessed by aligning reads to ribosomal database on SortMeRNA (Kopylova et al., 2012) (version 4.2.0), which reached up to ~10%/sample. Readcounts from BAMs, which were generated from the alignment to the primary ‘Russell’ genome, were performed using Subread (Liao et al., 2013) (version 1.5.3). Differential expression (DE) analysis on the raw readcounts was performed using three independent methods including voom (Law et al., 2014) (efit test, padj <0.05), EdgeR (Robinson et al., 2010) (quasi-likelihood test, FDR <0.05) and DESeq2 (Love et al., 2014) (padj <0.05).

**References**

Bandi, V.G., Carl (2020). Interactive Exploration of Genomic Conservation. In Proceedings of the 46th Graphics Interface Conference on Proceedings of Graphics Interface 2020 (GI’20). Canadian Human-Computer Communications Society, Waterloo, CAN.

Bolger, A.M., Lohse, M., and Usadel, B. (2014). Trimmomatic: a flexible trimmer for Illumina sequence data. Bioinformatics (Oxford, England) 30, 2114-2120. doi: 10.1093/bioinformatics/btu170

Danecek, P., Auton, A., Abecasis, G., Albers, C.A., Banks, E., Depristo, M.A., Handsaker, R.E., Lunter, G., Marth, G.T., Sherry, S.T., Mcvean, G., Durbin, R., and Group, G.P.A. (2011). The variant call format and VCFtools. Bioinformatics 27, 2156-2158. doi: 10.1093/bioinformatics/btr330

Dobin, A., Davis, C.A., Schlesinger, F., Drenkow, J., Zaleski, C., Jha, S., Batut, P., Chaisson, M., and Gingeras, T.R. (2012). STAR: ultrafast universal RNA-seq aligner. Bioinformatics 29, 15-21. doi: 10.1093/bioinformatics/bts635

Dunn, N.A., Unni, D.R., Diesh, C., Munoz-Torres, M., Harris, N.L., Yao, E., Rasche, H., Holmes, I.H., Elsik, C.G., and Lewis, S.E. (2019). Apollo: Democratizing genome annotation. PLOS Computational Biology 15, e1006790. doi: 10.1371/journal.pcbi.1006790

Garrison, E.P., and Marth, G.T. (2012). Haplotype-based variant detection from short-read sequencing. arXiv: Genomics.

Kim, D., Paggi, J.M., Park, C., Bennett, C., and Salzberg, S.L. (2019). Graph-based genome alignment and genotyping with HISAT2 and HISAT-genotype. Nature Biotechnology 37, 907-915. doi: 10.1038/s41587-019-0201-4

Kopylova, E., Noé, L., and Touzet, H. (2012). SortMeRNA: fast and accurate filtering of ribosomal RNAs in metatranscriptomic data. Bioinformatics 28, 3211-3217. doi: 10.1093/bioinformatics/bts611

Krzywinski, M., Schein, J., Birol, İ., Connors, J., Gascoyne, R., Horsman, D., Jones, S.J., and Marra, M.A. (2009). Circos: An information aesthetic for comparative genomics. Genome Research 19, 1639-1645. doi: 10.1101/gr.092759.109

Law, C.W., Chen, Y., Shi, W., and Smyth, G.K. (2014). voom: precision weights unlock linear model analysis tools for RNA-seq read counts. Genome Biology 15, R29. doi: 10.1186/gb-2014-15-2-r29

Li, H., and Durbin, R. (2009). Fast and accurate short read alignment with Burrows-Wheeler transform. Bioinformatics 25, 1754-1760. doi: 10.1093/bioinformatics/btp324

Li, H., Handsaker, B., Wysoker, A., Fennell, T., Ruan, J., Homer, N., Marth, G., Abecasis, G., Durbin, R., and Subgroup, G.P.D.P. (2009). The Sequence Alignment/Map format and SAMtools. Bioinformatics 25, 2078-2079. doi: 10.1093/bioinformatics/btp352

Liao, Y., Smyth, G.K., and Shi, W. (2013). The Subread aligner: fast, accurate and scalable read mapping by seed-and-vote. Nucleic Acids Research 41, e108-e108. doi: 10.1093/nar/gkt214

Love, M.I., Huber, W., and Anders, S. (2014). Moderated estimation of fold change and dispersion for RNA-seq data with DESeq2. Genome Biology 15, 550. doi: 10.1186/s13059-014-0550-8

Marçais, G., Delcher, A.L., Phillippy, A.M., Coston, R., Salzberg, S.L., and Zimin, A. (2018). MUMmer4: A fast and versatile genome alignment system. PLOS Computational Biology 14, e1005944. doi: 10.1371/journal.pcbi.1005944

Pilkington, S.M., Crowhurst, R., Hilario, E., Nardozza, S., Fraser, L., Peng, Y., Gunaseelan, K., Simpson, R., Tahir, J., Deroles, S.C., Templeton, K., Luo, Z., Davy, M., Cheng, C., Mcneilage, M., Scaglione, D., Liu, Y., Zhang, Q., Datson, P., De Silva, N., Gardiner, S.E., Bassett, H., Chagné, D., Mccallum, J., Dzierzon, H., Deng, C., Wang, Y.-Y., Barron, L., Manako, K., Bowen, J., Foster, T.M., Erridge, Z.A., Tiffin, H., Waite, C.N., Davies, K.M., Grierson, E.P., Laing, W.A., Kirk, R., Chen, X., Wood, M., Montefiori, M., Brummell, D.A., Schwinn, K.E., Catanach, A., Fullerton, C., Li, D., Meiyalaghan, S., Nieuwenhuizen, N., Read, N., Prakash, R., Hunter, D., Zhang, H., Mckenzie, M., Knäbel, M., Harris, A., Allan, A.C., Gleave, A., Chen, A., Janssen, B.J., Plunkett, B., Ampomah-Dwamena, C., Voogd, C., Leif, D., Lafferty, D., Souleyre, E.J.F., Varkonyi-Gasic, E., Gambi, F., Hanley, J., Yao, J.-L., Cheung, J., David, K.M., Warren, B., Marsh, K., Snowden, K.C., Lin-Wang, K., Brian, L., Martinez-Sanchez, M., Wang, M., Ileperuma, N., Macnee, N., Campin, R., Mcatee, P., Drummond, R.S.M., Espley, R.V., Ireland, H.S., Wu, R., Atkinson, R.G., Karunairetnam, S., Bulley, S., Chunkath, S., Hanley, Z., Storey, R., Thrimawithana, A.H., Thomson, S., David, C., Testolin, R., Huang, H., Hellens, R.P., and Schaffer, R.J. (2018). A manually annotated *Actinidia chinensis* var. *chinensis* (kiwifruit) genome highlights the challenges associated with draft genomes and gene prediction in plants. BMC Genomics 19, 257. doi: 10.1186/s12864-018-4656-3

Pilkington, S.M., Crowhurst, R., Hilario, E., Thomson, S., Le Lievre, L., and Brownfield, L. (2021). *Actinidia chinensis* Red5 genome assembly (version 2) and annotation files. *Zenodo* <https://doi.org/10.5281/zenodo.5717387>.

Prjdb8483, N.B. (2020). *Actinidia rufa* (nashi-kazura), Strain: Fuchu. https://www.ncbi.nlm.nih.gov/assembly/GCA_014362265.1.

Robinson, M.D., Mccarthy, D.J., and Smyth, G.K. (2010). edgeR: a Bioconductor package for differential expression analysis of digital gene expression data. Bioinformatics (Oxford, England) 26, 139-140. doi: 10.1093/bioinformatics/btp616

Su, W., Ou, S., Hufford, M.B., and Peterson, T. (2021). "A Tutorial of EDTA: Extensive De Novo TE Annotator," in Plant Transposable Elements: Methods and Protocols, ed. J. Cho. (New York, NY: Springer US), 55-67

Tang, W., Sun, X., Yue, J., Tang, X., Jiao, C., Yang, Y., Niu, X., Miao, M., Zhang, D., Huang, S., Shi, W., Li, M., Fang, C., Fei, Z., and Liu, Y. (2019). Chromosome-scale genome assembly of kiwifruit *Actinidia eriantha* with single-molecule sequencing and chromatin interaction mapping. GigaScience 8. doi: 10.1093/gigascience/giz027

Wang, Y., Tang, H., Debarry, J.D., Tan, X., Li, J., Wang, X., Lee, T.H., Jin, H., Marler, B., Guo, H., Kissinger, J.C., and Paterson, A.H. (2012). MCScanX: a toolkit for detection and evolutionary analysis of gene synteny and collinearity. Nucleic Acids Research 40, e49. doi: 10.1093/nar/gkr1293

Wu, H., Ma, T., Kang, M., Ai, F., Zhang, J., Dong, G., and Liu, J. (2019). A high-quality *Actinidia chinensis* (kiwifruit) genome. Horticulture Research 6, 117. doi: 10.1038/s41438-019-0202-y

Wu, T.D., and Watanabe, C.K. (2005). GMAP: a genomic mapping and alignment program for mRNA and EST sequences. Bioinformatics 21, 1859-1875. doi: 10.1093/bioinformatics/bti310
